# Voice Analysis to Differentiate the Dopaminergic Response in People With Parkinson's Disease

**DOI:** 10.3389/fnhum.2021.667997

**Published:** 2021-05-31

**Authors:** Anubhav Jain, Kian Abedinpour, Ozgur Polat, Mine Melodi Çalışkan, Afsaneh Asaei, Franz M. J. Pfister, Urban M. Fietzek, Milos Cernak

**Affiliations:** ^1^Center for Innovation and Business Creation at Technical University of Munich (UnternehmerTUM), Munich, Germany; ^2^Department of Neurology and Clinical Neurophysiology, Schön Klinik München Schwabing, Munich, Germany; ^3^xMint, Yalova, Turkey; ^4^Department of Mechatronics Engineering, Boğaziçi University, Istanbul, Turkey; ^5^Department of Data Science, Ludwig Maximilians Universität, Munich, Germany; ^6^Department of Neurology, University of Munich, Munich, Germany; ^7^Logitech Europe, École polytechnique fédérale de Lausanne - Quartier de l'Innovation, Lausanne, Switzerland

**Keywords:** Parkinson's disease, speech, voice, dopaminergic response, motor state

## Abstract

Humans' voice offers the widest variety of motor phenomena of any human activity. However, its clinical evaluation in people with movement disorders such as Parkinson's disease (PD) lags behind current knowledge on advanced analytical automatic speech processing methodology. Here, we use deep learning-based speech processing to differentially analyze voice recordings in 14 people with PD before and after dopaminergic medication using personalized Convolutional Recurrent Neural Networks (p-CRNN) and Phone Attribute Codebooks (PAC). p-CRNN yields an accuracy of 82.35% in the binary classification of ON and OFF motor states at a sensitivity/specificity of 0.86/0.78. The PAC-based approach's accuracy was slightly lower with 73.08% at a sensitivity/specificity of 0.69/0.77, but this method offers easier interpretation and understanding of the computational biomarkers. Both p-CRNN and PAC provide a differentiated view and novel insights into the distinctive components of the speech of persons with PD. Both methods detect voice qualities that are amenable to dopaminergic treatment, including active phonetic and prosodic features. Our findings may pave the way for quantitative measurements of speech in persons with PD.

## 1. Introduction

Parkinson's disease (PD) is a clinically highly variable neurodegenerative disorder. A wealth of information points to the paramount role of reduced dopaminergic neurotransmission in the nigrostriatal pathways for PD pathophysiology. Ultimately, neuronal activity in the basal ganglia and related circuits involved in motor control becomes dysfunctional, resulting in a characteristic motor phenotype with loss of movement amplitude, slowing of movement, and loss of automaticity (Hughes et al., 1992).

This loss in motor performance affects the voice in a very distinctive way. Persons with PD (PwPD) speak more softly, slur, may often hesitate in talking, have breathiness and hoarse voice quality, along with imprecise articulation (Logemann et al., 1978). Patients have typically shown to lose the natural inflections in speech which brings forth variations in the pitch and tone patterns, making their speech more monotonous. On the one hand, a cardinal symptom of PD is the slowness of movement, i.e., bradykinesia, and sometimes we see the slow speech. On the other hand, we observe hastened movements, especially in gait, termed festination, or speech, where typically PwPD can have hastened, festinating speech, which is very characteristic. Actually, slow bradykinetic speech is quite rare in PD. The typical patient speaks lower (hypophonic), huskier/hoarse, and interestingly, having a characteristic hastened speech, i.e., faster mode of speaking.

Given this abundance of easily accessible symptoms, it almost comes as a surprise that the standard clinical examination of PwPD, e.g., by using the Movement Disorder Society sponsored version of the Unified Parkinson's Disease Rating Scale (MDS-UPDRS), only uses one item (item III.1) for the evaluation of speech focusing on impairment and understandability, less on the symptomatology (Goetz et al., 2008). Indeed, the rich information resulting from voice alterations is rarely used for clinical decision-making, and often considered a disorder in itself, less a symptom of PD.

With progressing disease, speech pathology also deteriorates (Skodda et al., 2013), and may even become unresponsive to previous successful dopamine treatment (Tykalová et al., 2015). It is relevant to note that in later disease stages, non-motor symptoms, e.g., pooling of saliva in the mouth, become increasingly common and affect speech production (Muzerengi et al., 2007). Such observations have put forth the idea that speech production in PwPD may respond less convincingly to dopamine replacement therapy as other motor symptoms, or may even be completely not responsive to dopamine therapy.

So speech is a highly appropriate “motor behavior“ that is poorly represented in clinical assessment and decision making. Understanding speech by advanced data analytics methods could be instrumental for the evaluation of PwPD, e.g., for detection of therapy response or guiding personalized evaluation of patients.

Therefore in this work, we have looked at two distinctive machine learning-based speech analysis methodologies to assess the dopaminergic response by classification at the “ON” and “OFF” moments of PwPD. We have applied these techniques to data collected at the Schön Klinik München Schwabing, Germany, thereby testing out approaches.

## 2. Related Work

As dopamine can dramatically alleviate the motor symptoms in PD, there is a profound interest to quantify the effect of those medications. Currently, there exist no standardized methods to determine short-term and long-term medication's effects or side effects. Clinical ratings from experts regularly lack granularity and are provided at a maximum of four times per day (Erb et al., 2020). Imaging techniques such as the DaTSCAN are timely and costly and require the patient to accept radionuclide exposition (Booij et al., 1999). Recently, the use of machine learning to evaluate highly granular sensor data on the response of motor symptoms of PwPD to dopamine replacement therapy has gained wide attention (Pfister et al., 2020).

In previous studies, other researchers have investigated speech in PwPD using it as a paradigm for the motor phenotype of PD. They demonstrated clearly that voice/speech can provide an abundance of highly informative and distinguishing aspects for the evaluation of PwPD. For example, Orozco-Arroyave et al. (2016) show quantitative and visual distinctions of speech in PwPD against healthy controls.

Early explorations of Skodda et al. (2010) found a positive effect of dopaminergic stimulation on vowel articulation in individual patients diagnosed with mild PD, later confirmed by Okada et al. (2015). Overall, none of the examined phonation parameters: intonation, articulation, and speech velocity, improved significantly in the ON state, neither under short-term levodopa administration nor on stable dopaminergic treatment. Improvement of vowel articulation seen in individual patients supports the personalized approach we follow in our work. Speech measures of Poluha et al. (1998), duration of the vowels /i/, /u/, /æ/, the quadrilateral area produced by these vowels, and the slope of the diphthong /aɪ/ also did not show a significant trend across the levodopa cycle. Likewise, Santos et al. (2010) did not find a statistical difference in the analysis of the acoustic parameters in PwPD at the ON and OFF motor states. The considered parameters were as follows: fundamental frequency, jitter, shimmer, harmonic noise proportion, index of tremor, and voice quality. Besides, Skodda et al. (2011) reported no Levodopa administration effect on the fundamental frequency and the speech rate. Results of Brabenec et al. (2017) also confirm these claims: hypokinetic dysarthria in PwPD seems to be mainly related to non-dopaminergic deficits.

On the contrary, quantitative acoustic analyses of Rusz et al. (2016) revealed that speech disorders of PwPD tend to improve or remain relatively stable after the initiation of dopaminergic treatment. They appear to be related to the dopaminergic responsiveness of bradykinesia. Tykalová et al. (2015) found a strong positive correlation between the total cumulative dose of levodopa and the increased occurrence of dysfluent words and stuttering. Likewise, results from Im et al. (2019) indicate that levodopa medication can significantly affect speech dysfluency, primarily associated with the severity of the OFF medication. Speech analysis has been done on a standard Rainbow passage reading task.

Current studies on differentiating the ON and OFF medication states have mixed or contradictory results. Conclusions of Ho et al. (2008) well characterize the previous mixed results research: speech response to dopaminergic treatment shows a consistent tendency for increased loudness and faster rate during the ON state, but accompanies by a greater extent of intensity decay with pitch and articulation remained unchanged. The dopaminergic treatment effect on the final acoustic product of speech may or may not be advantageous, depending on individual patients' existing speech profile. All those conclusions support our proposed personalized approach to voice analysis of PwPD.

Although Im et al. (2019) conclude that the comparison of the PD participants in the ON state vs. OFF state conditions did not reach statistical significance, the last explorations performed recently, based on machine and deep learning by Norel et al. (2020); Pompili et al. (2020) reported higher accuracies of ON and OFF classification. Norel et al. (2020) analyzed voice of PwPD describing a picture, speaking reverse counting, and diadochokinetic rate speech tasks. Mel Frequency Cepstral Coefficients (MFCCs) were the most informative. However, the study reported only classification accuracy without standard statistical measures such as sensitivity and specificity. Also, their problem formulation, performing (“ON”–“OFF”) vs. (“OFF”–“ON”) instead of “ON” vs. “OFF” classification is questionable. Besides, the interpretability of the used speech representation is limited, as individual MFCCs bins represent specific frequency bandwidths without interpretation to speech perception and production. Pompili et al. (2020) devised an automatic speech processing system with deep learning-based ON–OFF classifiers. The eGeMAPS features of Eyben et al. (2015), composed of basic acoustic speech parameters and a storytelling speech task, obtained the best performance. Comparing to previous systems, the authors designed speaker-dependent models, a limitation that might be important in clinical praxis. The accuracy of the speaker-independent model explored in Pompili et al. (2020) was limited up to 65% that justified their adoption of the speaker-dependent approach.

Our work aims to explore the feasibility of detecting clinically meaningful dopamine effects in the speech of PwPD with deep learning methods. Deep learning requires lots of data; can it be successfully applied to limited speech training data? On the other hand, instance-based machine learning comes with a great interpretation power that tries to mimic human cognition in the analysis.

## 3. Data and Protocols

### 3.1. Ethics and Setup

This study was reviewed by the Ethical board from the Technical University of Munich (No 234/16S). The data for this project were collected by an expert hospital involved in PD care—the Schön Klinik München Schwabing, Munich, Germany. Prior to data acquisition, all patients involved gave written informed consent to the study procedures, and to pseudonymized storage of voice recordings and further speech analyses.

### 3.2. Participants

In-house PwPD were recruited from October to December 2017 in the Schön Clinic München Schwabing. Patients were asked to participate and examined in a quiet exam room by a standardized speech protocol (see below). We report age, disease duration, MDS-UPDRS motor score III, and the speech item III.1.

### 3.3. Clinical Procedures

#### 3.3.1. Speech Protocol

The speech recording protocol consisted of isolated and connected speech. Doctors specifically designed seven different speech protocols to highlight and bring forth the impairment in articulation.

In the first speech protocol, **Digits**, the patients were asked to count till 10 twice in German. To record the protocol **Months**, the patients were asked to name the months in a calendar year from January to December twice. The **Vowels** task consists of the patients repeating the English vowels /a/-/e/-/i/-/o/-/u/ three times. To further stress upon their articulation, the test continued with the **Pataka** task, where the patients were asked to repeat the following words three times: /pataka/, /pakata/, /petaka/, and /pekata/ (diadochokinetic evaluation). The patients were requested to repeated the following German tongue twisters twice for the **Twisters** task: “Liebe Lilly Lehmann,” “Dritte berittene Kavalleriebrigade,” and “Schleimig schuppende Schellfischflossen.” The patients were given a short reading task as an additional task, **Read**. We used the German reading text from Orozco-Arroyave et al. (2016). For the final speech task **Monologue**, the patients were asked to follow the Cookie Theft description test and were asked to speak about it for approximately 45 s. The test is described in Cummings (2019). For consistency, the same image was shown to all participating patients.

#### 3.3.2. Data Acquisition

Speech raw data (.wav files) were recorded using a Rode NT1-A microphone and a Steinberg UR22 MK2 audio interface with a sampling frequency of 44.1 kHz and 32 floating-point sample encoding.

The average length of each recording session was 4 min; participants were asked to follow two subsequent recording sessions. For validation purposes, each recording was videotaped. Data were manually segmented according to the speech task.

### 3.4. Cohort

The Munich study cohort consisted of 16 PD patients (10 female, six male) with an average age of 66 ± 7 yrs (mean ± SD) and a mean disease duration of 11 ± 5 yrs. The mean motor score according to part III of MDS-UPDRS was 32 ± 14 assessed before levodopa intake and 21 ± 11 30 min after levodopa intake, resulting in a mean pre-post-levodopa difference (delta UPDRS) of 12 ± 5. Concerning item III.1 of the MDS-UPDRS, we saw a mean score of 1.93 before medication intake, and a mean score of 1.27 30 minutes after the intake, respectively. All the results are reported using the cohort of 14 patients. Table 1 provides the detailed UPDRS rating for the ON and OFF states for each patient along with other metadata such as Age and Gender.

**Table 1:** Unified Parkinson's disease rating scale ratings for patients in the Schön Klinik data.

**ID**	**Gender**	**Age**	**III Total ON**	**III Total OFF**	**III.I ON**	**III.I OFF**
1	F	53	26	39	2	2
2	F	62	18	34	1	2
3	F	68	8	15	1	1
4	F	67	21	40	0	1
5	M	73	18	26	1	2
6	F	68	4	10	0	0
7	M	73	34	50	2	3
8	M	74	31	53	3	4
9	F	55	28	36	1	1
10	M	66	19	35	3	4
11	F	70	6	14	0	1
12	F	76	28	39	1	2
13	M	66	11	21	0	1
14	M	70	41	48	3	4

### 3.5. Feature Extraction

All available speech recordings were downsampled to 16 kHz with 16-bits sample encoding and chunked into 10-s-long segments. The time-frequency representation of each segment was obtained using a complex short-time Fourier transform (STFT, 25 ms window length with 7.5 ms overlap, 201 frequency bins), resulting in a 201 × 571 matrix. The magnitude of each frequency channel was normalized using the mean and standard deviation obtained from the training data.

To obtain phone attribute posterior features from the speech segments, we use an open-source phonological vocoding platform (Cernak and Garner, 2016). These features are also known as distinctive and phonological features. Linguistic and neurocognitive studies recognize them as the essential and invariant representation used in temporal speech organization. Their properties are explored in details by Cernak et al. (2016, 2018).

The phonological analysis starts with a short-term analysis of speech, which consists of converting the speech signal into a sequence of acoustic feature vectors composed of the conventional MFCCs. The Mel scale is a perceptual scale often used in speech signal processing. Then, the phone attribute posterior probabilities are estimated for each frame. Each probability is computed independently using a binary classifier based on deep NNs (DNNs) and trained with one phonological class vs. the rest. Finally, the MFCCs sequence is transformed into a sequence of the phone attribute vectors. The features can be further quantized or compressed using sparse coding that relies on the phonological features' structured sparsity.

**Figure 1:**
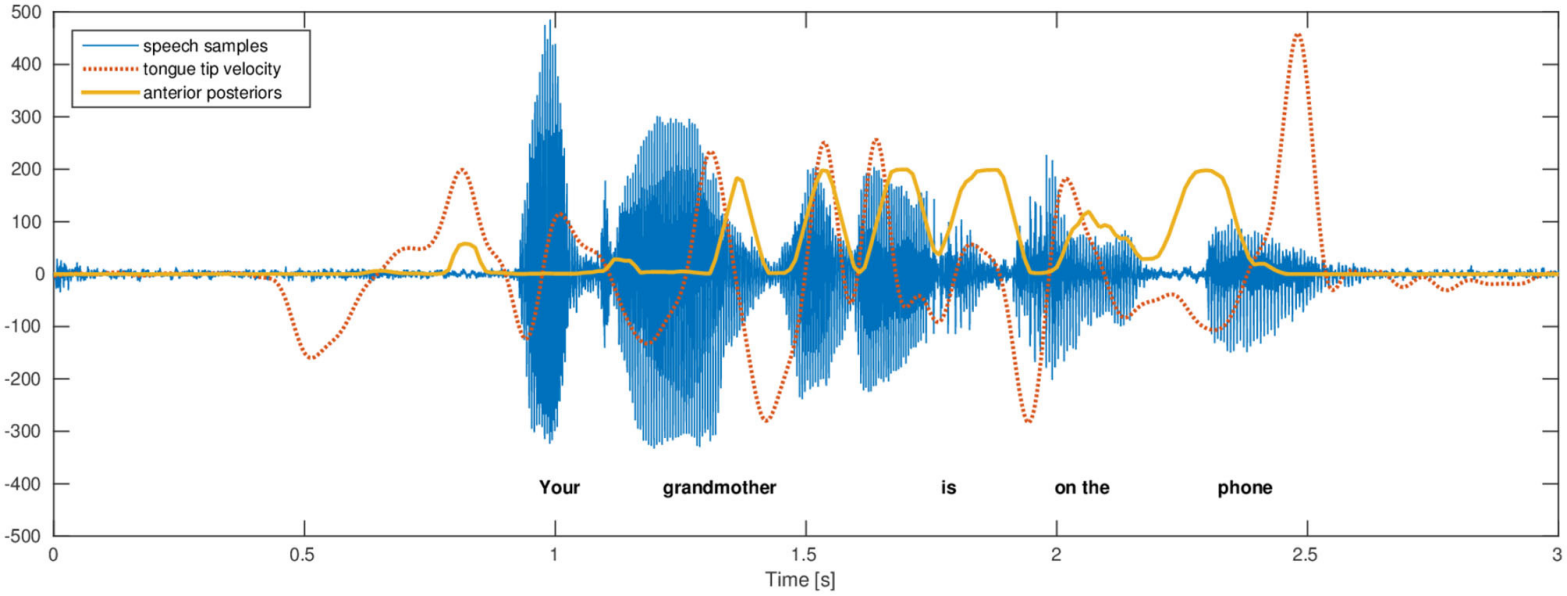
This figure, from Cernak et al. (2016), illustrates the variation of the electromagnetic articulography tongue tip velocity (vertical direction with respect to the occlusal plane) and phone-attribute anterior posterior feature. The trajectories have the same number of maximums and their relation is evident. A more asynchronous relation toward the end of utterance is caused by asynchronous relations of fast tongue tip movements causing an obstruction in the mouth and generated acoustics.

The phone attribute posterior features are directly interpretable, as illustrated by an example in Figure 1. The anterior posteriors estimated by the DNNs are highly correlated with the variation in electromagnetic articulography tongue tip velocity. This correlation showcases the relevance of these features and their interpretability.

## 4. Methodology

Motivated by Pompili et al. (2020), we hypothesize that a deep learning-based approach may find better data-driven models between voices of ON and OFF states. However, we want to focus on speaker-independent modeling. Section 4.1 describes the proposed deep learning approach. Its interpretation is limited, and thus section 4.2 introduces the PAC-based classification that offers an insight into the modeled problem. Section 4.3 describes a variant of deep learning-based method devoted to personalized speech assessment.

### 4.1. Deep Neural Network Based ON/OFF Classification

**Figure 2:**
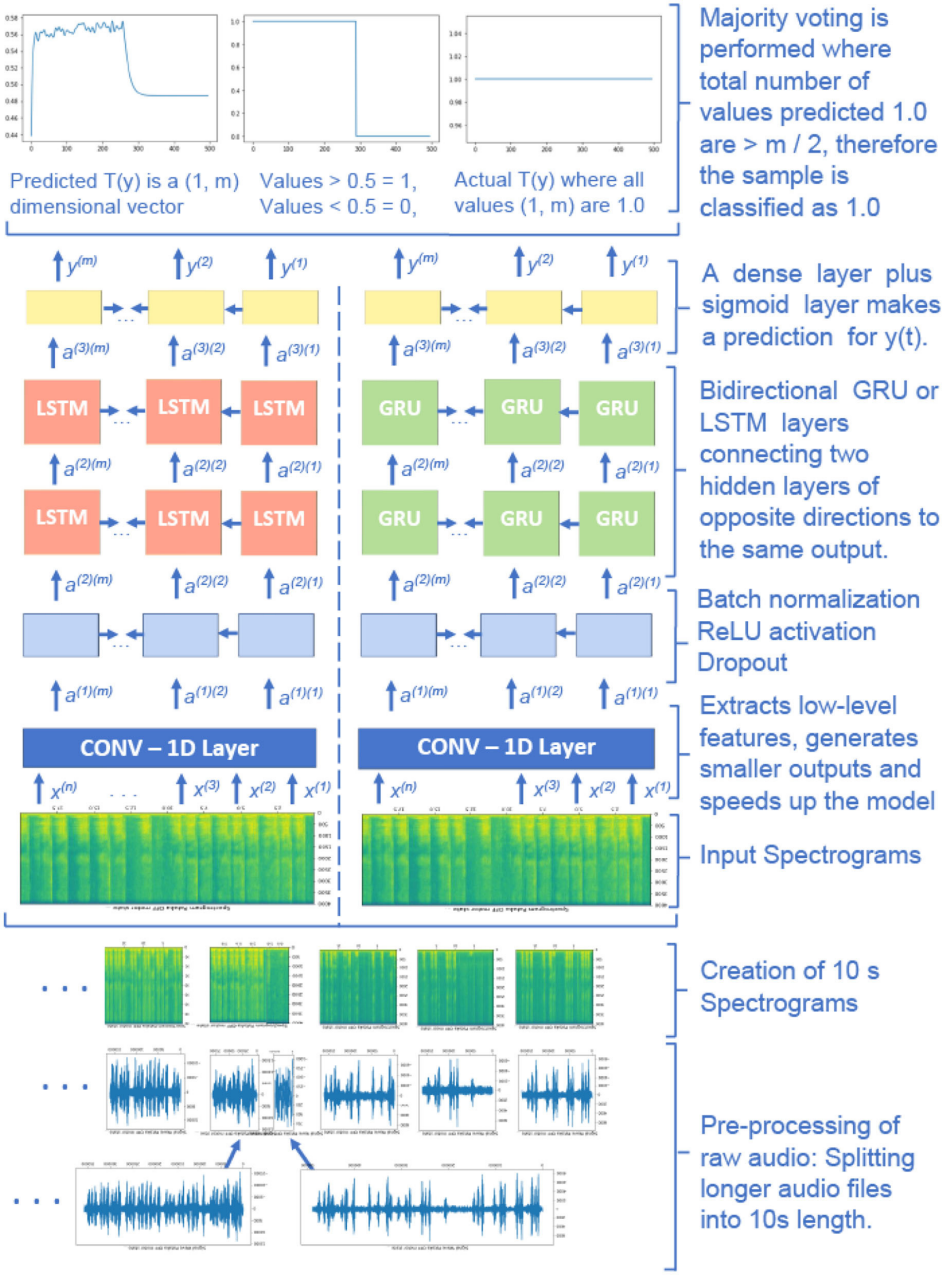
The deep learning workflow diagram. Convolutional part of the model extracts relevant distinctive ON-OFF regions, recurrent part analyses those regions and models time dependencies, and the dense layers find arithmetic relations between the analyzed regions and actual ON-OFF motor states.

All audio training files recorded with the same motor state are concatenated together and then cut to 10-s long segments. However, the testing files are not combined, as we have followed utterance-level evaluation. For the recordings in the test set, 10-s long segments are split into two 5 s parts, the former part used as validation and the latter for testing. We selected a Convolutional-Recurrent Neural Network (CRNN) architecture, which has shown excellent results previously in audio and speech classification tasks. The deep learning workflow diagram in Figure 2 shows the pre-processing (feature extraction) of audio data, the CRNN architecture (pattern learning), and final post-processing (decision making).

The input to the neural network architecture is 10-s long audio that is represented by the number of time steps *n* and the number of frequency bins *f* in each time step. The input is an (*n, f*) dimensional vector, and the values of *n* and *f* depend on the hyper-parameters that are selected to create the spectrograms. The target values are 0 and 1 for the OFF and ON states, respectively. The target values are repeated in each time step of a particular recording.

The first layer of the deep learning architecture consists of a 1−*D* convolutional layer that extracts lower-level features and speeds up the model. It contains 196 filters, a kernel size of 15, and strides of (4, 4) for the time and frequency dimensions. Thus, the output time steps are downsampled to *m*, which is smaller than the input *n*. Two subsequent stacked bidirectional Long Short-Term Memory (LSTM) or gated recurrent unit (GRU) layers have 128 units. This form of deep learning allows the output layer to get information from the past (backward) and future (forward) states simultaneously and can provide additional context to the network. Dropout with the rate of 0.8 and batch normalization layers are used after each recurrent layer. The final time-distributed dense layer with the sigmoid activation function outputs *Ty*, a (1, *m*) dimensional vector where the model returns predictions between 0 and 1 for each unit of *Ty*.

The output values between 0 and 1 are later converted to either 0 or 1 depending on a selected threshold value. An average threshold value of 0.44 is selected on the validation set. The final prediction is performed using majority voting of the neural network's estimated values on each 10 s sample. For example, if more than *m*/2 units are predicted as ON, the whole audio is predicted as ON and vice versa. All speech protocols are considered in the training and testing.

### 4.2. Phone Attribute Posteriors Codes Based ON/OFF Classification

Deep neural networks are used to capture phone attributes characterized by 20 distinct features derived from Chomsky and Halle (1991), plus the *silence* feature. The posterior attributes are represented as their corresponding posterior probabilities obtained during feature extraction of the speech segments. These features relate to the phonation and articulation of speech, which have proven to be useful for clinical decision making.

The proposed solution belongs to instance-based machine learning, as no model is trained here, and knowledge is captured in instances of the features. The phone-attribute posterior probabilities can be characterized in terms of binary codebooks by quantizing them using the 1-bit quantization level. Posterior probabilities below 0.5 are normalized to 0 while probabilities above 0.5 are normalized to 1. Phone-attribute posteriors indicate the physiological posture of the articulary system and the quantization step helps us identify the prominent active postures involved in articulation. As PD affects the brain, which in turn makes it difficult for the patient to articulate words, these quantized posterior probabilities help in the identification and isolation of the patients who have not received medication.

Each quantized binary sequence is referred to as a code. All the binary sequences pertaining to one class are accumulated together to create a codebook. In other words, a codebook is a set of unordered codes. For binary sequence matching, unique codebooks are required, which can be compared with the test sample's codebook. For this reason, we find unique ON and OFF codebooks—such that the intersection of both the codebooks is the null set. The codebooks are discriminative features that represent their corresponding motor stats, and they enable the classification of these states. The underlying premise of constructing unique quantized codebooks is that the codebook uniquely represents each class. The active phonological components for each class are universal and can be found in samples relating to others.

For the classification of any patient into their respective motor state, quantized codebooks are created for each speech protocol. Each code in the codebook is matched with the ones present in the ON and OFF codebook. The particular speech sample is classified with a motor state with more matches with its corresponding codebook.

As the number of unique permissible patterns is small, there are rare cases where the training dataset contains few unique codes in each codebook. In such cases, direct matches with the ON and OFF codebook may not exist. Thus, we take advantage of binary pattern matching techniques.

(1) $$\begin{array}{r} S_{JACCARD}=\frac{a}{a+b+c} \end{array}$$

For this particular use case, we have found that Jaccard distance achieves the best results. The Jaccard Similarity score can be computed using equation 1. For two binary sequences, *a* denotes the number of times both the sequences have a value 1 for the same phone-attribute feature. The term *b* represents the number of times the first sequence has a value 1, and the second has a value 0 for the same feature. Similarly, *c* can be computed by calculating the occurrences of 0 in the first sequence and 1 in the second sequence for the same position in the pattern.

As the final step, a majority voting is done on the class predictions of the available speech protocol to predict the patient's motor state correctly.

### 4.3. Personalized Speech Assessment

In personalized assessment, the audio file for each protocol for each patient is divided into validation and test sets. We utilized the predictions from the validation section of the speech recordings to find personalized speech protocols for each patient. Personalized protocols are defined as the ones that can successfully classify both the ON and OFF motor states. In the testing stage, only these personalized speech protocols are utilized for the assessment of the patients. Note that the trained model in the personalized assessment is still speaker independent. The personalized assessment is obtained using the same CRNN model introduced in section 4.1, but as per the protocol and thus personalized. We denote this approach as personalized-CRNN (p-CRNN).

In our experimentation, we realized that the personalized speech assessment is not possible for the PAC-based approach given the extremely small validation and testing sets of a few seconds of data. The PAC-based system relies heavily on the previous features' instances to find matches with the ON and OFF quantized codebooks. Given the relatively small data size, it yields inaccurate results or cases where there is no match found between the speaker's quantized features and the codebooks. Thus, to assess the patients in a personalized manner using a PAC-based approach, there is a need for larger amounts of data, especially two or more recordings per protocol for each patient. However, given the higher interpretability of this approach, it may attract medical practitioners' attention.

## 5. Results

We have followed leave-one-out cross-validation for all the experiments. The data corresponding to one patient is utilized for testing, and the data for the remaining patients are used for training. The reported results are the average of all patients.

Table 2 shows overall classification results. The CRNN model achieves an average accuracy of 80% over 16 different models that are trained using the leave-one-out cross-validation method. Data belonging to each speaker is removed from the training set and divided into two parts for testing and validation. This is repeated for all other speakers and model metrics such as accuracy, precision, recall, and specificity are calculated for each model as well as average values for all models.

**Table 2:** Overall classification accuracy for the proposed approaches: Phone-Attribute Codebook (PAC), Convolutional-Recurrent Neural Network (CRNN), and personalized CRNN (p-CRNN).

**Method**	**Accuracy**	**Sensitivity/Recall**	**Specificity**	**Precision**
PAC	0.73	0.69	0.77	0.75
CRNN	0.80	0.71	0.89	0.85
p-CRNN	0.82	0.86	0.78	0.80

Patients perform seven different speech tasks. The performance of the models has been measured for each task as well as their overall performance. It has been observed, for example, that while the model for Speaker 1 achieves precision, recall, and specificity of 1.00 for vowels task, it gives values below 0.50 for other tasks. In contrast, while the model for Speaker 2 achieves precision, recall, and specificity values above 0.60 for monologue and read tasks and 1.00 for the other tasks, it gives values equal to zero for the vowels task. Model for Speaker 12 gives values above 0.80 for twisters and monologue tasks but does not perform well on other tasks. Like the model in Speaker 1, Speaker 13 performs well only for a single task (monologue).

The fact that trained models perform well for some tasks, but not others can indicate that personalized speech tasks might improve overall model performance. Thus, we devised a p-CRNN approach that outperformed CRNN one in terms of accuracy. The results for the p-CRNN approach indicate the advantage of the speaker-dependent personalized assessment of the speech. This further validates our hypothesis that since the disease uniquely affects each patient, this approach is needed to capture medication's response more accurately.

The PAC-based method achieved lower classification performance, but their phone-attribute posterior features provide a high level of interpretability as the human cognitive system inspires them. The phone-attribute features are class conditional probabilities of 21 phonological classes derived from 21 different DNNs. A phone is a speech segment that possesses distinct physical or perceptual properties and serves as the basic phonetic speech analysis unit. Different phones combined together represent the speech segment phoneme such as classes *continuant, back, vocalic*, and *voice* compose the phoneme/ɑ/. This indicated a structural dependency between different classes, which is of paramount interest.

A two-sample paired *t*-test was performed to evaluate whether the phone attribute features varied in the two motor states. The evaluation compared their values for the 21 features across the two medication states. The results showed significant differences in values in the ON and OFF motor states for most of the features. The features *fricative, silence*, and *continuant* had high t-score values of −18.91, 15.83, and −14.26, respectively (*p* < 0.001). This indicated that these features show significant variation in the ON and OFF states and are useful for their distinction. While the phone-attribute features *retroflex* and *velar* had a lower *t*-score value of −1.02 and −1.65, respectively (*p* < 0.001). Significant statistical differences of the phone-attribute features in the ON and OFF states motivated us to design interpretable classification using the features.

**Figure 3:**
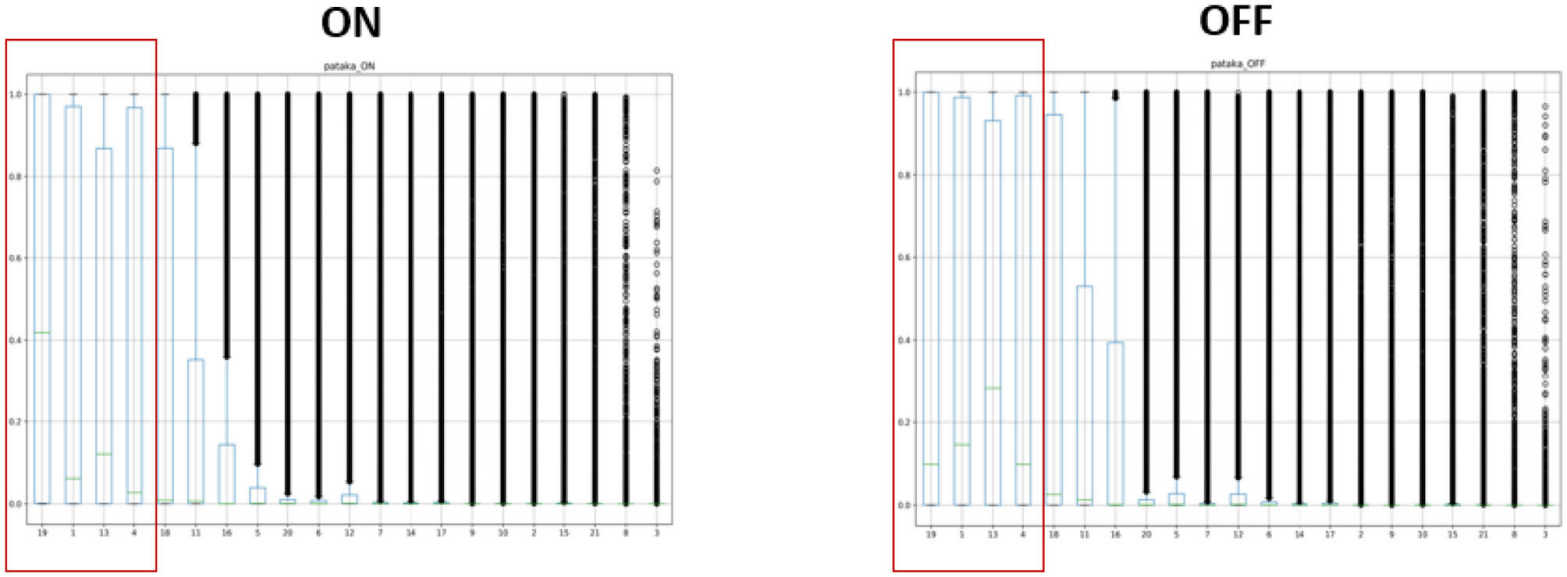
An illustrated example of spoken “pataka”; a small subset of features that characterizes the speech: silence, continuant, tense, and voiced are the only phone attributes that showed ON and OFF variation.

**Figure 4:**
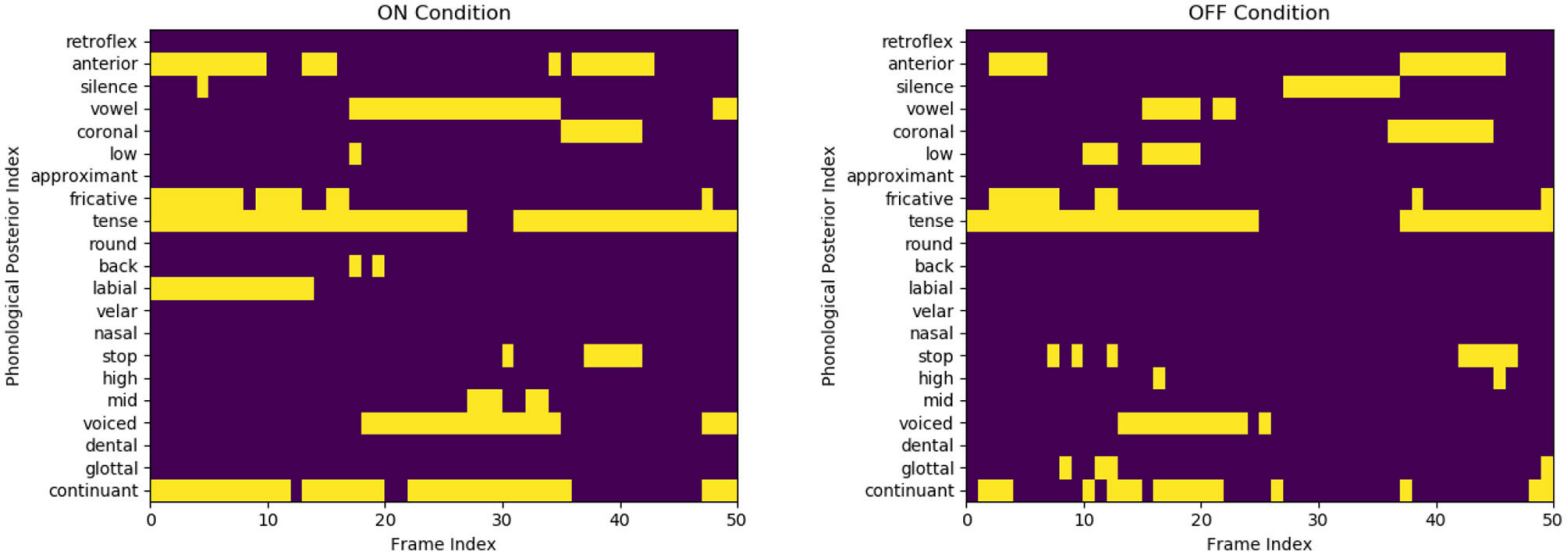
Representation of few binary codes for the ON and OFF states for the 21 phone attributes. Although the speaker and the speech content are the same (speaker three saying /pataka/), we observe a substantial variation in the phone attribute quantification. The OFF variant is visually more sparse and misses some distinctive features, like *labial* in initial /p/ production.

Figure 3 of the *pataka* sub-task shows that only 4 out of the 21 phonological classes categorize the speech. However, as shown in Table 3, these phonological classes are dependent on each other. This structural dependency between different classes is utilized for the classification of the motor states. The table clearly shows how pairs such as *high* and *coronal* have a high 2D correlation in the ON state and similarly *continuant* and *tense* have a high correlation in the OFF state. By creating binary codebooks, we are exploiting these structural-dependent features to represent the motor states uniquely. This allows the creation of unique binary codebooks, as shown in Figure 4. It is the example sequence of phone attribute posteriors of the same speech sounds produced by the same speaker in the ON and OFF states.

**Table 3:** Correlation between some phone-attribute posterior features for the *pataka* sub-task depicting the importance of structural dependency.

**State**	**Dependent pairs**	**2-D correlation**
ON	(*high, coronal*)	+0.97
	(*mid, silence*)	−0.88
OFF	(*continuant, tense*)	+0.92
	(*tense, silence*)	−0.93

## 6. Discussion

This study compares three automated machine learning methods to evaluate people's speech with PD in different functional motor states resulting from their fluctuating dopaminergic treatment. Thus, we are able to reproduce previous seminal results from others concerning the dopaminergic response in PwPD related to the patients' individual speech profiles (Ho et al., 2008; Skodda et al., 2010). The paper presents two basic speech analysis methodologies—instance, machine learning-based, and deep learning-based. We introduce a novel personalized speech assessment approach that employs a speaker-independent model using particular evaluation speech tasks.

The differentiation of variant motor states is of fundamental importance in evaluating the motor function of PwPD and currently provides the basis for interpreting most therapies in advanced disease stages. Our three variant methods demonstrate convincing and realistic accuracies ranging from 0.73 to 0.83 to achieve the task of differentiation of ON from OFF motor states. Implementing a leave-one-out cross-validation for all the experiments ascertains that our results are robust, even using a small dataset.

Our p-CRNN approach uses 10-s input audio segments easily sampled from the patients' recordings. Using the first 10 s of recordings or repeating shorter segments to obtain the required sample length, we allow for easy and economically feasible sampling of critical health data. Another benefit of the speech-based assessment is the equipment's negligible cost coupled with the procedure's complete safety. Thus, the personalized models' results provide a path to interpret small quantities of speech data to recognize the individual level's motor state. Indeed, in the personalized model that we introduce to the literature for the first time, we provide a safe and objective differentiation of the patient's functional dopaminergic state.

Among the comprehensive set of protocols used in other similar automatic analyses, such as in Orozco-Arroyave et al. (2018), followed to record the patients' speech before and after medication, different protocols have proven to show different levels of variations in ON and OFF states. In contrast, a few of the protocols demonstrated a steady behavior not affected by medication. So the distinctive capability of different evaluation protocols is shown to be a speaker-dependent parameter.

PD's motor syndrome affects every patient in a unique and highly individual fashion. Besides, as the impairment in articulation may not be reflected in every spoken word, a personalized approach becomes essential in analyzing the differentiation introduced to the speech by variant motor states, i.e., defining dopaminergic deficit. For this reason, we propose a speaker-dependent evaluation methodology that selects relevant speech protocols that highlight the impairment in speech for the given patient. In contrast to Pompili et al. (2020), the p-CRNN method uses speaker-independent models.

The methods applied for speech analyses may hold further keys to relevant clinical information. The speech protocols **pataka** and **vowels** show a higher level of variability in the two motor states for the PAC-based approach, with the pataka subtask being the most effective and yielding the higher accuracy. Especially for the speech task pataka, the higher variability can be attributed to the complexity in pronunciation of the word.

**Figure 5:**
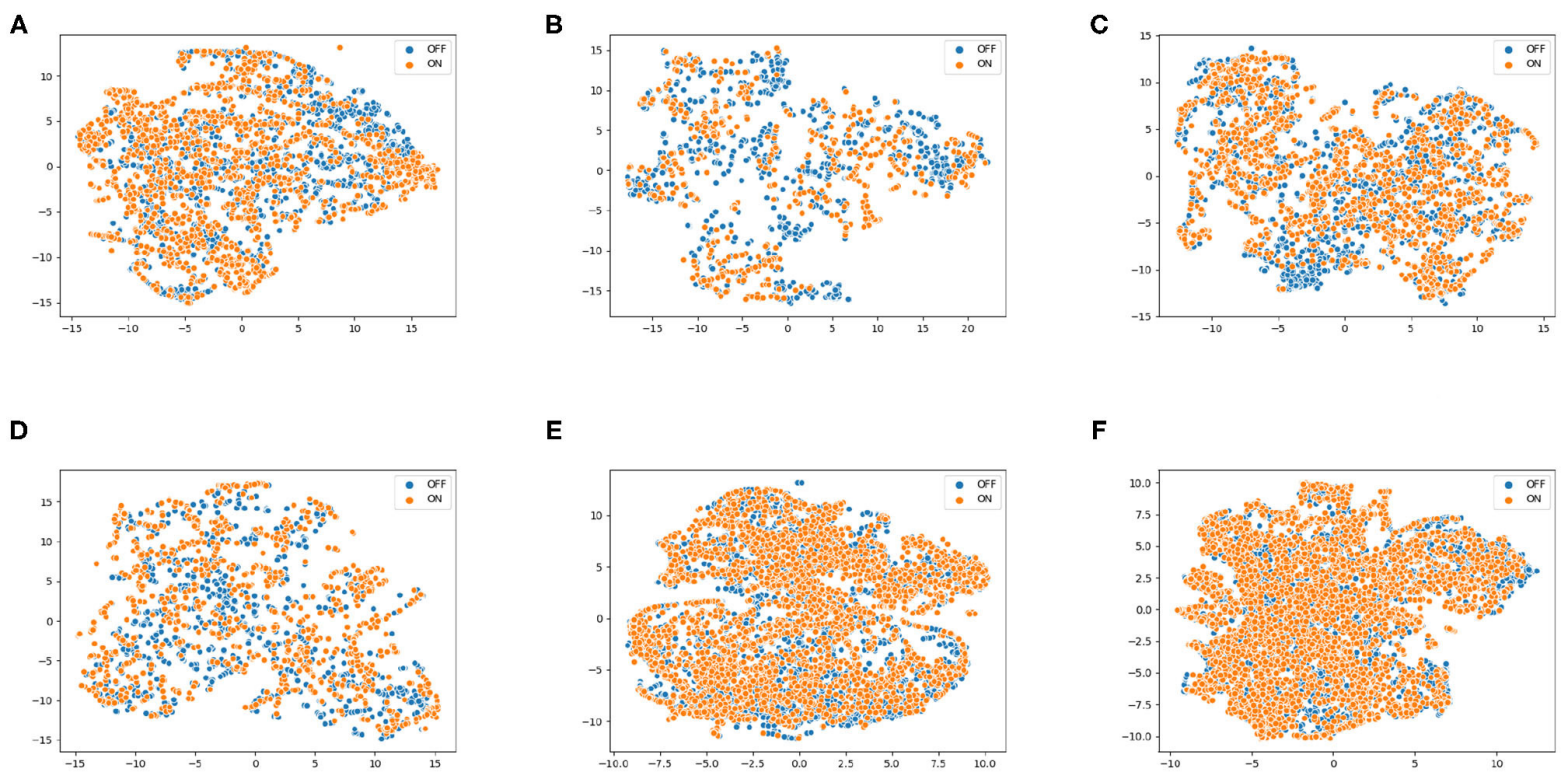
t-SNE plots using phone-attribute posterior features for various speech protocols, **(A)** pataka, **(B)** vowels, **(C)** twisters, **(D)** digits, **(E)** monologue, and **(F)** read, illustrating the differences in their ability to distinguish the and OFF. Experimentation show similar results where monologue and read protocols perform poorly, while pataka and vowels allow better distinction.

Figure 5 representing t-SNE plots for the various speech protocols further highlights the difference in different protocols' ability to distinguish the two classes. **Monologue** and **read** are often misclassified, which can be observed from their t-SNE plots: The overlapping ON and OFF classes indicate the lower level of separability, which is coherent with the PAC-based approach's protocol-wise accuracy scores.

One methodological issue concerns the definition of our predicted classes, the ON and OFF motor states. Only in far advanced patients, sudden motor state changes within seconds are observed, which constitutes the basis for distinct ON and OFF motor states, as described in Marsden and Parkes (1976). In less advanced PD stages, the OFF motor state occurs more slowly, e.g., in the morning after dopamine depletion overnight or as wearing-off before the next dopamine medication intake. This was the case for the PwPD examined in this project, where we had a practical OFF motor state that does not correspond to complete dopamine depletion. This might also help to explain the difficulty of achieving higher accuracies beyond 0.8. Novel data from people with PD and suddenness of ON-OFF fluctuations may provide further information about dopamine's role in PD speech.

Currently, the evaluation of speech contributes only a small section in the overall motor state evaluation of PwPD. This is represented in the single item referring to speech in the 33 item motor rating score most often used to evaluate PwPD by Goetz et al. (2008). This project delineates several interesting pathways for further speech and voice usage in detecting and analyzing the digital phenotype of PwPD and their response to medications. Thus, the deep neural networks' confidence scores might help quantify and standardize short and long-term dopaminergic medication responses.

## 7. Conclusion and Future Work

Speech is a highly complex motor behavior that encodes a rich combination of variations in motor output involving various neuronal centers of the brain. Computational models that enable quantification of speech and interpretation of its attributes as digital biomarkers might be instrumental for the objective early, and even prodromal detection of relevant symptoms. The models presented here, using a personalized adaptation, might help to provide a trough of insights for the analysis of impaired speech in PwPD.

This paper shows that speech can be highly instrumental for the clinical evaluation of relevant questions for people with PD by detection of the dopaminergic effects on individual speech. This may guide the medical practitioners to the personalized evaluation of the most individual human motor task, and the effect of the most relevant medication for PD.

We have used an elaborate voice recording situation with a studio-like setting. In the future, our methods might be expanded to include data collected from mobile situations or devices, e.g., automatically sampled, or from telephone calls. This could help to apply the method in a more continuous fashion.

For a more reliable interpretation of results, the current database will need to be enlarged. Doing so will be necessary to evaluate the personalized approach for the PAC-based method, and will require having multiple recordings for the same speech protocol. More data would mean a reduced risk to over-fit, and could entail more variations of clinical concern.

## Data Availability Statement

The patients of this proof-of-concept study, in accordance to National and European Data protection laws, did not consent to publication of their sensor data in open repositories. The data that support the findings of this study are available from the corresponding author, UF, upon reasonable request.

## Ethics Statement

The studies involving human participants were reviewed and approved by The Schön Klinik München Schwabing, Parzivalplatz 4, 80804 Munich, Germany. The patients/participants provided their written informed consent to participate in this study. This project was approved by the ethical board of the Technical University of Munich (TUM) (No. 234/16S) on June 30, 2016.

## Author Contributions

AJ and OP have equal contributions to the submission. UF and MC are seniors in this project. All authors contributed to the article and approved the submitted version.

## Conflict of Interest

MMÇ was employed by the company xMint. MC was employed by the company Logitech. The remaining authors declare that the research was conducted in the absence of any commercial or financial relationships that could be construed as a potential conflict of interest.
